# The Prognostic Value of ADAMTS-13 and von Willebrand Factor in COVID-19 Patients: Prospective Evaluation by Care Setting

**DOI:** 10.3390/diagnostics11091648

**Published:** 2021-09-09

**Authors:** Giovanni Tiscia, Giovanni Favuzzi, Antonio De Laurenzo, Filomena Cappucci, Lucia Fischetti, Donatella Colaizzo, Elena Chinni, Lucia Florio, Giuseppe Miscio, Angela Pamela Piscitelli, Mario Mastroianno, Elvira Grandone, on behalf of the CSS COVID-19 Group

**Affiliations:** 1 Thrombosis and Haemostasis Unit, Fondazione IRCCS “Casa Sollievo della Sofferenza”, 71013 San Giovanni Rotondo, Italy; g.tiscia@operapadrepio.it (G.T.); g.favuzzi@operapadrepio.it (G.F.); antonio.delaurenzo@gmail.com (A.D.L.); filomena.cappucci@libero.it (F.C.); l.fischetti@operapadrepio.it (L.F.); d.colaizzo@operapadrepio.it (D.C.); e.chinni@operapadrepio.it (E.C.); 2 Unit of Neurology, Fondazione IRCCS “Casa Sollievo della Sofferenza”, 71013 San Giovanni Rotondo, Italy; l.florio@operapadrepio.it; 3 Unit of Transfusion Medicine and Clinical Pathology, Fondazione IRCCS “Casa Sollievo della Sofferenza”, 71013 San Giovanni Rotondo, Italy; g.miscio@operapadrepio.it; 4 Unit of Internal Medicine, Fondazione IRCCS “Casa Sollievo della Sofferenza”, 71013 San Giovanni Rotondo, Italy; ap.piscitelli@operapadrepio.it; 5 Scientific Direction, Fondazione IRCCS “Casa Sollievo della Sofferenza”, 71013 San Giovanni Rotondo, Italy; m.mastroianno@operapadrepio.it; 6 Ob/Gyn Department of the First I.M. Sechenov Moscow State Medical University, 119435 Moscow, Russia

**Keywords:** COVID-19, endothelium, von Willebrand factor, ADAMTS-13, fatalities

## Abstract

Background: Endothelial dysfunction, coupled with inflammation, induces thrombo-inflammation. In COVID-19, this process is believed to be associated with clinical severity. Von Willebrand factor (VWF), and a disintegrin and metalloproteinase with thrombospondin motifs 13 (ADAMTS-13), are strong markers of endothelial dysfunction. We evaluated the impact of the VWF/ADAMTS-13 fraction on COVID-19 severity and prognosis. Materials and methods: A cohort study including 74 COVID-19 patients, with 22 admitted to the intensive care unit (ICU) and 52 to the medical ward (MW), was carried out. We also evaluated, in a group of 54 patients who were prospectively observed, whether variations in VWF/ADAMTS-13 correlated with the degree of severity and routine blood parameters. Results: A VWF:RCo/ADAMTS-13 fraction above 6.5 predicted in-hospital mortality in the entire cohort. At admission, a VWF:RCo/ADAMTS-13 fraction above 5.7 predicted admission to the ICU. Furthermore, the VWF:RCo/ADAMTS-13 fraction directly correlated with C-reactive protein (CRP) (Spearman r: 0.51, *p* < 0.0001) and D-dimer (Spearman r: 0.26, *p* = 0.03). In the prospective cohort, dynamic changes in VWF:RCo/ADAMTS-13 and the CRP concentration were directly correlated (Spearman r, *p* = 0.0014). This relationship was significant in both groups (ICU: *p* = 0.006; MW: *p* = 0.02).Conclusions: The present findings show that in COVID-19, the VWF/ADAMTS-13 fraction predicts in-hospital mortality. The VWF/ADAMTS-13 fraction may be a helpful tool to monitor COVID-19 patients throughout hospitalization.

## 1. Introduction

The number of published papers on the new viral disorder COVID-19 has now exceeded the threshold of 80,000. Several studies focused on prognostic aspects, and a large portion of them reported an association between COVID-19 severity and significant changes in hemostasis and coagulation markers. 

Several studies found that D-dimer predicts mortality, as it progressively increases in those who develop the mostsevere clinical spectrum [1,2,3]. It has been hypothesized that the mechanisms for the increased D-dimer in COVID-19 may be related to the virus life cycle. The apoptotic process targets the endothelial cells, which results in activation of coagulation and, in turn, in the increase in D-dimer [4].

Previous reports showed a significant increase in the markers of endothelial dysfunction in COVID-19. For instance, von Willebrand factor (VWF) levels and high-molecular-weight multimers are mainly observed in the most-critical patients [5,6]. On the other hand, hyper-inflammation may also contribute to the endothelial dysfunction in COVID-19 [7].

Therefore, there is a strong relationship between endothelial injury, the inflammation process, activation of the coagulation cascade, and the severity of the disease. Consistent with this, several studies documented an association between reduced activity levels of ADAMTS-13 and mortality [8,9].We previously found that ADAMTS-13, at hospital admission, predicts mortality in COVID-19 patients [10]. Indeed, as for septic patients, low ADAMTS-13 levels are inversely correlated with VWF and a poor prognosis [11].

The aims of the present study were as follows: (1) to compare the VWF/ADAMTS-13 fraction in COVID-19 patients according to the care setting, and explore the relationship between these parameters and inflammatory markers; (2) to prospectively evaluate whether, and to what extent, the VWF/ADAMTS-13 fraction is predictive of mortality.

## 2. Materials and Methods

### 2.1. Study Methodology and Setting

We enrolled 74 patients consecutively observed between 1 March and 30 September 2020, with a laboratory-confirmed SARS-CoV-2 infection (i.e., RT-PCR according to the protocol established by the WHO). In all cases, we collected at baseline all relevant demographic/clinical information and routine panel blood parameters. Exclusion criteria were as follows: age below 18 years, pregnancy, confirmed VWF disease. 

We compared findings from patients admitted to the intensive care unit (ICU) to those observed in patients hospitalized in the medical ward (MW). Furthermore, we tested a possible relationship of VWF and ADAMTS-13 with a poor prognosis. 

In a group of 54 patients prospectively observed, we also evaluated whether variations [calculated as Δ (delta: in-hospital changes)] value on blood samples collected after at least five days from the baseline) in VWF/ADAMTS-13 correlated with the degree of severity and routine blood parameters. The study was approved by our ethics committee and carried out in accordance with the lastly amended Helsinki Declaration.

### 2.2. Sample Preparation and Plasma-Based Determinations

Venous blood was collected into 0.1 vol. 0.129 M sodium citrate and platelet-poor plasma was prepared by centrifugation of 10 min at 1600× *g* and stored in aliquots. VWF:RCo and VWF:Ag were measured, using an automated latex immunoassay in human citrated plasma on ACL TOP^®^ coagulation systems (HemosIL^®^). ADAMTS-13 activity was measured, using a chromogenic ELISA assay (TECHNOZYM^®^ ADAMTS-13 activity ELISA kit, Technoclone, Vienna, Austria) as previously described [12].

### 2.3. Data Analysis

Categorical variables were described as numbers and percentages. Normal variables were summarized as means and standard deviations, and non-normal variables as medians and interquartile range (IQR). We used the χ^2^ test, Fisher’s exact test, or Mann–Whitney U test to compare differences where appropriate according to data distribution, examined by means of the D’Agostino & Pearson test. Significance was set at *p* values below 0.05. 

## 3. Results

### 3.1. Demographic/Clinical Information and Laboratory Data

Among the 74 patients recruited (all patients were Caucasian), 22 were hospitalized in the ICU and 52 in the MW. Table 1 depicts the demographic and clinical information of the entire cohort. The patients admitted to the ICU were significantly younger than those admitted to the MW, with a higher proportion of males in the ICU (77% vs. 50%). Furthermore, the two groups were similar with regard to the pre-existing co-morbidities and fatalities (Table 1). As far as routine blood parameters are concerned, the white blood cells (WBC), neutrophil-to-lymphocyte ratio (NRL), lactate dehydrogenase (LDH), direct bilirubin, alanine aminotransferase (ALT), activated partial thromboplastin time (aPTT), fibrinogen, D-dimer, and factor VIII were significantly different according to the care setting (Table 1). 

### 3.2. VWF/ADAMTS-13 Fraction by Care Setting 

The VWF activity levels were significantly higher in the patients admitted to the ICU than those measured in the patients admitted to the MW, whereas VWF:antigen (VWF:Ag) and ADAMTS-13 activity were not significantly different (Table 2). However, the VWF/ADAMTS-13 fraction was significantly higher in the ICU patients than in those admitted to the MW [VWF:Ag/ADAMTS-13 fraction: 5.7 (5.8) vs. 2.9 (1.8), *p* = 0.03; VWF:RCo/ADAMTS-13: 6.0 (2.6) vs. 3.4 (2.7), *p* < 0.0001—Mann–Whitney U test] (Table 2). 

**Figure 1:**
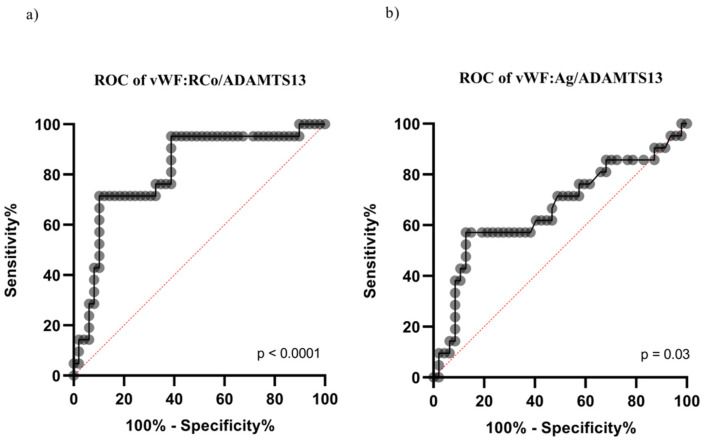
ROC curve for the prediction of ICU admission according to VWF:RCo/ADAMTS-13 and VWF:Ag/ADAMTS-13 fractions. (**a**) ROC curve for the prediction of ICU admission according to VWF:RCo/ADAMTS-13; (**b**) ROC curve for the prediction of ICU admission according toVWF:Ag/ADAMTS-13.

Furthermore, VWF:RCo predicted the admission to the ICU [area under the curve (AUC) = 0.80 (*p* < 0.0001), cut-off: 450 U/dL]. Similarly, the VWF:RCo/ADAMTS-13 and VWF:Ag/ADAMTS-13 fractions were associated with ICU admission [VWF:RCo/ADAMTS-13: AUC = 0.81 (range: 0.70–0.92), *p* < 0.001 (Figure 1a); VWF:Ag/ADAMTS-13 fraction: AUC = 0.66 (range: 0.51–0.81), *p* = 0.03, Figure 1b]. Values ≥ 5.7 of VWF:RCo/ADAMTS-13 and 5.2 of VWF:Ag/ADAMTS-13 predicted the ICU admission. 

### 3.3. Correlation between VWF/ADAMTS-13 and Routine Blood Parameters: Data from the Prospective Cohort

At hospital admission, we found, in the whole group (*n* = 74), a direct relationship between the VWF:RCo/ADAMTS-13 fraction and either D-dimer (Spearman r: 0.26, *p* = 0.03) and or C-reactive protein (CRP) (Spearman r: 0.51, *p* < 0.0001). Furthermore, CRP and D-dimer showed a direct correlation (Spearman r: 0.31, *p* = 0.01). 

**Figure 2:**
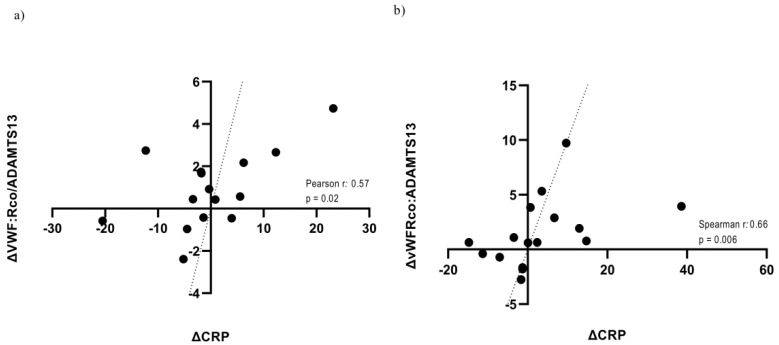
Correlation between in-hospital trajectories of VWF:RCo/ADAMTS-13 and CRP according to care setting. (**a**) Correlation between in-hospital trajectories of VWF:RCo/ADAMTS-13 and CRP in the MW; (**b**) Correlation between in-hospital trajectories of VWF:RCo/ADAMTS-13 and CRP in the ICU. CRP: C-Reactive Protein.

In the prospective cohort of 54 patients, dynamic changes in the VWF:RCo/ADAMTS-13 fraction directly correlated with variations (expressed as Δ value) in CRP concentration (Spearman r = 0.54, *p* = 0.0014). This relationship was statistically significant in both groups [MW: *p* = 0.02 (Figure 2a); ICU: *p* = 0.006 (Figure 2b)]. 

Lastly, the VWF:RCo/ADAMTS-13 fraction did not significantly correlate with serum albumin (Spearman test: *p* = 0.19). 

### 3.4. VWF/ADAMTS-13 and Mortality

**Figure 3:**
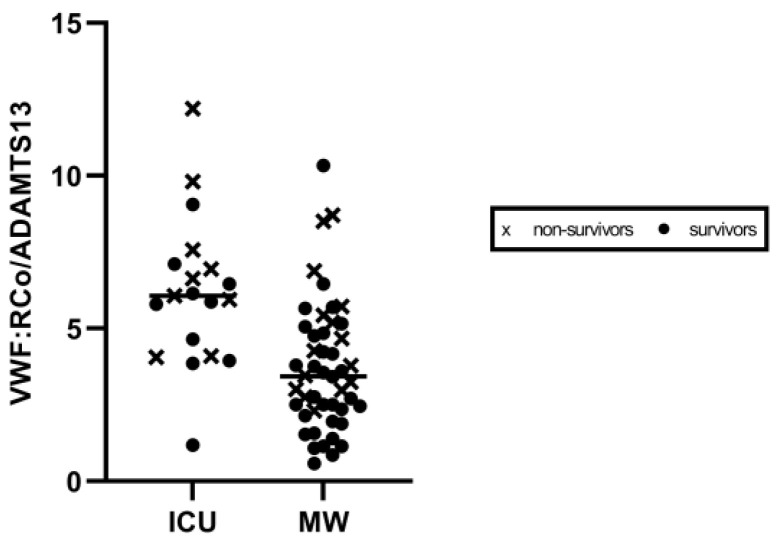
Scatter plot of VWF:RCo/ADAMTS-13 values according to care setting. Values in ICU and medical ward are shown on the left and right side, respectively. ICU: Intensive Care Unit. MW: Medical Ward.

**Figure 4:**
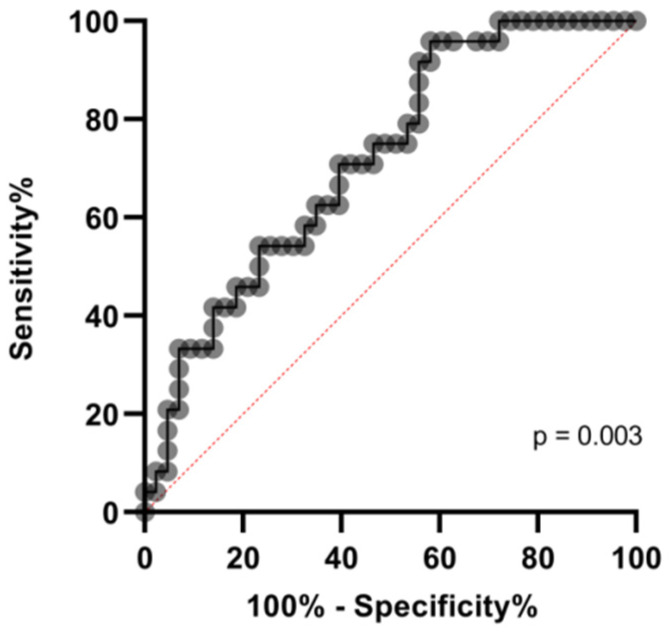
ROC curve for the mortality prediction according to VWF:RCo/ADAMTS-13 fraction.

Most fatalities, occurring either in the ICU (6/10, 60%) as well as in the MW (10/16, 62.5%), showed the VWF:RCo/ADAMTS-13 fraction to be above the median values (reported in Table 2) (Figure 3). A cut-off of 6.5 in VWF:RCo/ADAMTS-13 predicted in-hospital mortality with AUC: 0.71, (*p* = 0.003), as demonstrated by receiving operative curve (ROC) analysis (Figure 4). 

The D-dimer concentration was significantly higher in non-survivors compared to that calculated in survivors (3461 ng/mL vs. 913.5 ng/mL, Mann–Whitney U test: *p* = 0.007). In addition, the ADAMTS-13 (normal range: 0.40–1.30 U/mL) value was significantly lower in the fatalities (non-survivors: 60.0 U/dL vs. survivors: 80.0 U/dL; Mann–Whitney test: *p* = 0.007). 

We did not find any association between other routine laboratory markers and mortality. 

## 4. Discussion

In the present study, we confirm and extend the previous findings [8,9,10,13,14]. We show that the VWF/ADAMTS-13 fraction predicts ICU admission. These findings support the concept that in patients who need intensive care, endothelial activation may be more pronounced. 

The exposure to a variety of triggers makes the endothelium dysfunctional. Sepsis is one of the factors inducing alterations in endothelial biomarkers [15]. In this context, VWF (increase), ADAMTS-13 (reduction), and their fraction (increase) are good predictors of the clinical conditions in ICU septic patients [16,17]. As is well recognized, the endothelium stores (in intracellular granules called Weibel–Palade bodies) and—after its activation—secretes VWF [18]. Notably, there is evidence supporting the hypothesis that COVID-19 is an endothelial disease, as the endothelium plays a pivotal role in the processes counteracting the infectious agent [19]. The clinical picture of patients described here met no criteria for disseminated intravascular coagulopathy (i.e., the platelet count and fibrinogen concentration were within the normal ranges). Thus, the findings from the present research are consistent with the hypothesis that COVID-19 seems to be a specific coagulopathy, mainly characterized by endothelial dysfunction [20,21]. 

As expected, we found a higher D-dimer concentration in the patients admitted to the ICU than in those admitted to the MW. This may reflect a more pronounced pro-thrombotic state in ICU patients. The D-dimer concentration shows a significant association with COVID-19 severity, reaching the highest values in the patients admitted to the ICU and in those requiring mechanical ventilation [22,23]. The present results are consistent with the previous findings, as the D-dimer levels observed in the ICU patients were more than 10-fold higher than those observed in the MW patients.

We found a direct correlation between the VWF:RCo/ADAMTS-13 fraction and either CRP or D-dimer. Furthermore, we showed a direct relationship between CRP and D-dimer measured at hospital admission. Interestingly, throughout the entire period of hospitalization, the VWF:RCo/ADAMTS-13 and CRP trajectories correlated. This relationship was statistically significant in both the care settings (MW: *p* = 0.02; ICU: *p* = 0.006). Taken together, these findings are consistent with a strong interplay between inflammation, coagulation, and endothelial dysfunction, supporting the hypothetical pathogenesis of thrombo-inflammation in COVID-19 [20]. CRP is a highly sensitive, acute-phase marker, especially in septic patients [24].An increase in its gene expression is observed during inflammatory states; during infections, specifically, interleukin-6 (IL-6) is its main inductor [25]. A relationship between coagulation and inflammation has been previously shown; actually, tissuefactor (TF) plays a central role through the activation of protease-activated receptors (PARs) [26], which, in turn, stimulate cytokine release [27]. Of mention, the cytokine storm, observed in the course of COVID-19, is associated with induction of CRP gene over-expression in later disease stages [28]. It is plausible that the VWF/ADAMTS-13 fraction indicates the progression of endothelial and multi-organ damage. Moreover, endothelium dysfunction is likely to occur in the later stages of the disease [28], and its variations in the VWF/ADAMTS-13 fraction might be the main laboratory marker. 

We did not find a persistent relationship over time between dynamic variations in VWF:RCo/ADAMTS-13 and D-dimer. It is known that COVID-19 patients taking anticoagulants show modifications of D-dimer concentration [29]. All our patients were administered with low-molecular-weight heparin throughout hospitalization, as recommended by the international guidelines. Therefore, we cannot exclude that the pharmacological approach could have influenced this relationship.

A fraction of VWF:RCo/ADAMTS-13 corresponding to 6.5 predicted fatalities independently of the care setting; the higher the fraction was, the higher the risk was. The VWF:RCo/ADAMTS-13 fraction was a good marker of prognosis in our setting. This finding is consistent with the previous observation that micro-vascular occlusions and unfavorable outcomes in infectious diseases might be associated with hyper-activation and the consumption of vascular mediators, such as VWF and ADAMTS-13, remarking the concept that an imbalance in these parameters indicates, in general, a TMA picture [30]. Furthermore, our findings might be also in line with the hypothesis that seriously ill patients with COVID-19 might show a TMA clinical picture [31].

In our series, serum albumin was well below the normal values (2.4 g/L). However, hypoalbuminemia did not seem to affect the disease severity and clinical outcome, which was likely because of the limited number of patients. Albumin is physiologically bound within the glycocalyx of the endothelium, thus contributing to the stability of the layer [32]. It has been hypothesized that hypoalbuminemia is associated with ischemia, likely via endothelium damage and, in turn, activation of the coagulation cascade. Thus, low albumin levels might be associated with thrombotic events in acutely ill patients, reflecting pro-inflammatory or hypercoagulable states [33]. In COVID-19 patients, as well as in other critical settings, low serum albumin is associated with poor outcomes [34,35,36], and albumin infusion has been suggested to be an effective treatment to improve prognosis in COVID-19 [37,38]. 

The relatively limited number of patients and the design of the study (i.e., single-center investigation) are the limitations of the present study. Therefore, we must be cautious in generalizing the findings. However, the confirmation of the main findings in the prospective cohort confers robustness to present the data. 

## 5. Conclusions

Predicting the course of a COVID-19 patient’s disease after hospitalization is essential to improve treatment. The VWF/ADAMTS-13 fraction may assist clinicians in the assessment of care intensity in this setting. Our findings add knowledge to understanding COVID-19, contributing to widening the data collection on prognostic biomarkers that are potentially helpful in identifying patients with a worse prognosis.

## Figures and Tables

**Table 1:** Comparison of demographic/clinical information and blood parameters in patients admitted to ICU or medical ward.

Variables	All, *n* = 74	ICU, *n* = 22	Medical Ward, *n* = 52	*p* Value
Age, years, (IQR)	68.0 (22.0)	63.0 (15.2)	69.0 (19.7)	0.03
Male, *n* (%)	43 (58)	17 (77)	26 (50)	0.02
Deaths, *n* (%)	26 (35.0)	10 (45.5)	16 (30.7)	0.19
Invasive ventilation, *n* (%)	11 (15)	11 (50)	0 (0)	<0.0001
Non-invasive ventilation, *n* (%)	30 (40.5)	11 (50)	19 (36.5)	0.31
Diabetes, *n* (%)	15 (20)	6 (27.3)	9 (17.3)	0.35
Hypertension, *n* (%)	36 (48.5)	11 (50)	25 (48)	0.90
Chronic Kidney Disease, *n* (%)	11 (15)	6 (27.3)	6 (11.5)	0.16
Cardiovascular disease, *n* (%)	19 (25.5)	5 (22.7)	14 (27)	0.77
No. of co-morbidities, *n* (%)				
0	35 (47)	7 (32)	18 (34.5)	0.99
1	22 (29.7)	6 (27.0)	16 (31.0)	0.31
2	14 (19)	6 (27.0)	8 (15.5)	0.33
3	7 (9.5)	0	7 (13.5)	0.09
4	4 (5.5)	2 (9.1)	2 (3.8)	0.50
5	2 (2.7)	1 (4.5)	1 (2)	0.50
Red Blood Cells, 10^12^/L, (IQR)	4.3 (1.3)	4.4 (1.0)	4.3 (1.4)	0.91
White Blood Cells, 10^9^/L, (IQR)	7.4 (5.6)	10.6 (6.5)	5.8 (4.5)	0.03
Neutrophil-to-Lymphocyte Ratio, (IQR)	7.4 (15.4)	23.0 (24.0)	4.7 (6.6)	<0.0001
Hemoglobin, g/dL, (IQR)	12.5 (3.9)	12.6 (3.6)	12.3 (3.9)	0.78
Platelet count, 10^9^/L, (IQR)	242 (152)	254 (103)	238 (183)	0.48
Lactate Dehydrogenase, U/L, (IQR)	251.5 (240.0)	556.0 (512.0)	222.0 (94.5)	<0.0001
C-Reactive Protein, mg/dL, (IQR)	5.6 (9.0)	6.5 (11.4)	4.9 (8.1)	0.15
Direct bilirubin, mg/dL, (IQR)	0.1 (0.1)	0.2 (0.3)	0.1 (0.1)	0.0003
Indirect bilirubin, mg/dL, (IQR)	0.2 (0.2)	0.2 (0.2)	0.2 (0.2)	0.27
Aspartate transaminase, U/L, (IQR)	34.0 (27.0)	38.0 (32.5)	31.0 (24.5)	0.06
Alanine transaminase, U/L, (IQR)	31.0 (35.7)	32.0 (45.5)	30.0 (30.0)	0.045
Creatinine, mg/dL, (IQR)	0.9 (0.9)	0.7 (0.8)	1.0 (1.1)	0.05
Serum albumin, g/dL, (IQR)	2.4 (0.6)	2.4 (0.4)	2.4 (1.2)	0.87
Prothrombin Time, INR, (IQR)	1.1 (0.1)	1.1 (0.2)	1.1 (0.2)	0.58
Partial thromboplastin time, s, (IQR)	24.8 (4.4)	22.8 (5.0)	25.3 (4.0)	0.03
Fibrinogen, mg/dL, (IQR)	544.0 (351.5)	462.5 (260.2)	636.0 (238.5)	0.0083
D-dimer, ng/mL, (IQR)	1364.0 (5051.5)	11,936.0 (31,569.0)	1069.0 (2258.0)	<0.0001
Factor VIII, %, (IQR)	117.8 (68.0)	146.2 (55.8)	95.2 (61.9)	0.0032

**Table 2:** Comparison of VWF and ADAMTS-13 in patients admitted to ICU or medical ward.

Variables	All, *n* = 74	ICU, *n* = 22	Medical Ward, *n* = 52	*p* Value
vWF:Ag, U/dL, (IQR)	223.2 (207.4)	494.1 (362.8)	222.8 (30.4)	0.07
vWF:RCo, U/dL, (IQR)	324.1 (265.5)	439.8 (195.3)	266.5 (196.8)	<0.0001
ADAMTS-13, U/dL, (IQR)	80.0 (30.0)	70.0 (20.0)	80.0 (30.0)	0.83
vWF:Ag/ADAMTS-13 fraction, (IQR)	3.1 (4.0)	5.7 (5.8)	2.9 (1.8)	0.03
vWF:RCo/ADAMTS-13 fraction, (IQR)	4.1 (3.3)	6.0 (2.6)	3.4 (2.7)	<0.0001

## Data Availability

The data presented in this study are available on request from the corresponding author.
